# Supplementary material for: Drug and single-cell gene expression integration identifies sensitive and resistant glioblastoma cell populations
Source: Nat Commun. 2026 Jan 7;17:99. doi: 10.1038/s41467-025-67783-5 (PMC12780015; doi:10.1038/s41467-025-67783-5)
Supplement: Supplementary file 1 — Supplementary Information File [file 41467_2025_67783_MOESM1_ESM.pdf]

Supplementary Information:

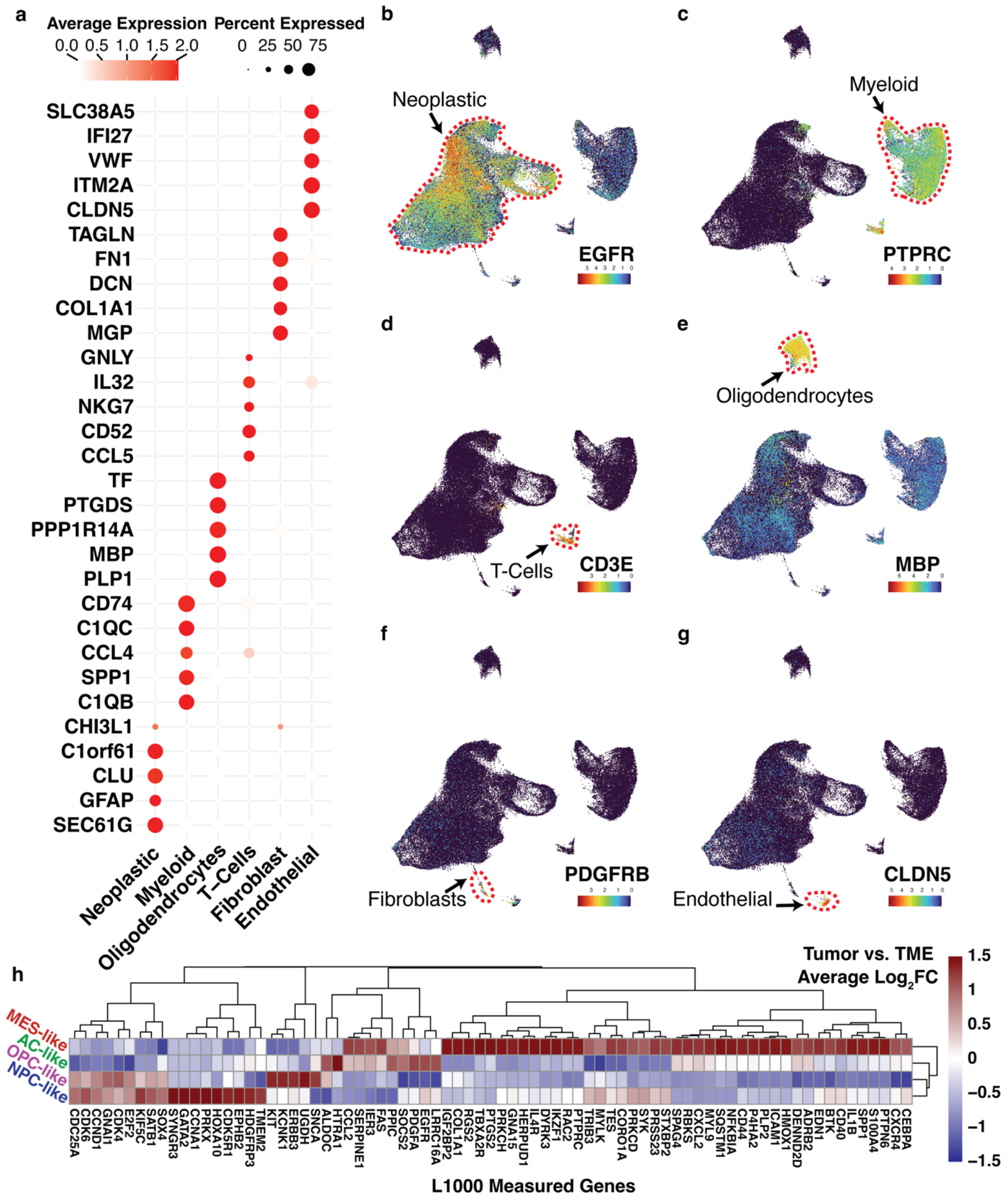

Supplementary Figure S1: Single-cell RNA sequencing reveals distinct non-neoplastic cell types present in both newly diagnosed and recurrent GBM. a. Dot plot of top marker expression of discrete cell types. The dot size encodes the percentage of cells within each class (cell type) with detected expression, while the color depicts the mean expression level across all cells within each class (cell type). **b-g.** UMAP plots depicting the scaled expression of the cell type-specific markers EGFR **(b)**, PTPRC **(c)**, CD3E **(d)**, MBP **(e)**, PDGFRB **(f)**, and CLDN5 **(g)**. **h.** Heatmap depicting relative differential expression of L1000-measured genes within pseudo-bulk disease signatures calculated using MAST (p.adj < 0.05). Heatmap color represents the scaled log_2FC relative to each GBM transcriptional state, calculated for each transcriptional state vs non-neoplastic cell populations within the scRNAseq data.

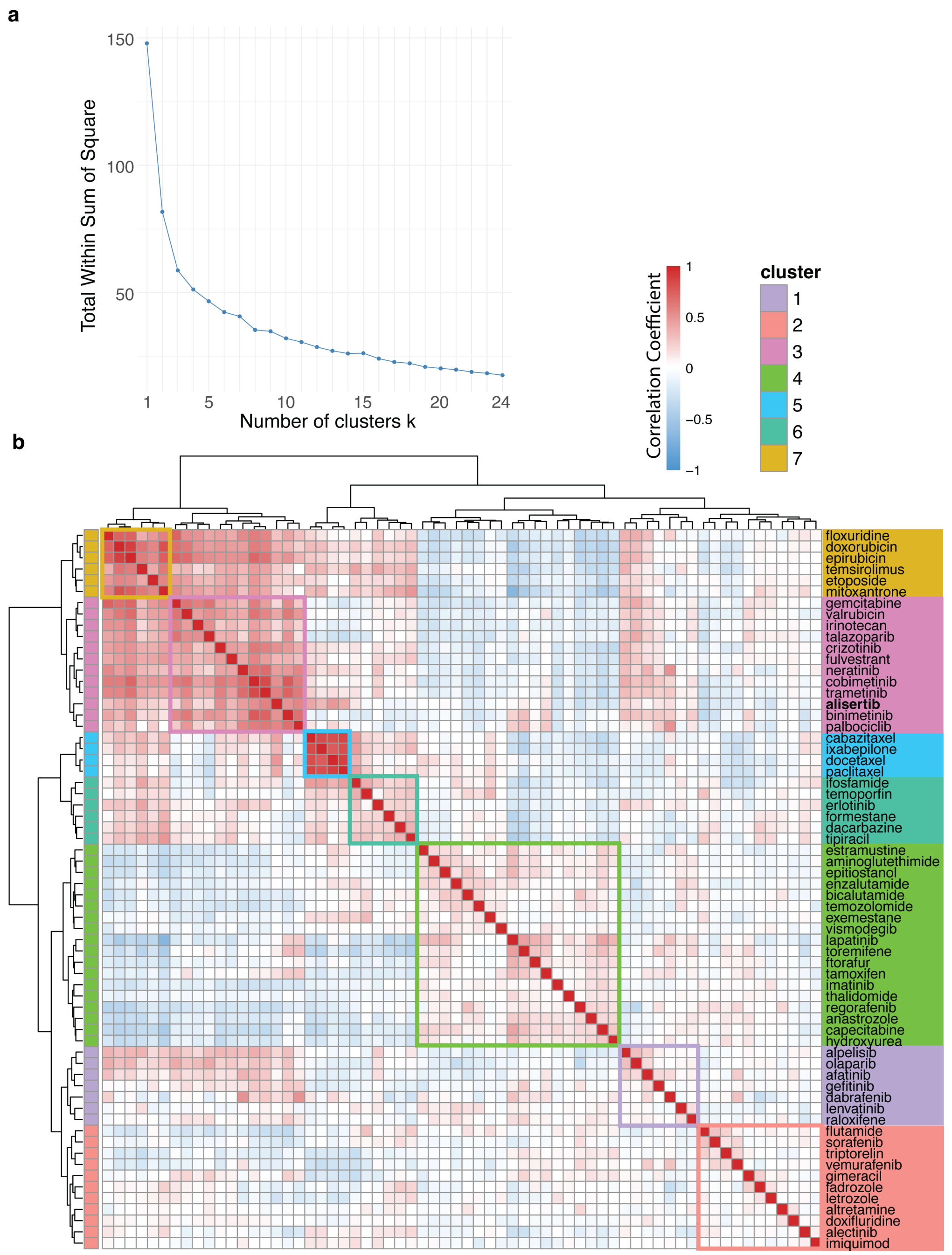

Supplementary Figure S2: FDA-approved oncology drugs can be clustered on their L1000 TCS connectivity to GBM tumor cell expression. a. Elbow plot depicting the within-cluster sum of squares by number of clusters k for connectivity values of 64 clinical oncology drug TCSs with all GBM tumor cells from 11 patients in the scRNAseq atlas. **b.** Correlation matrix heatmap depicting pairwise Spearman correlations of 64 clinical oncology drugs based on their connectivity values to all GBM tumor cells from the 11 patients in the scRNAseq atlas.

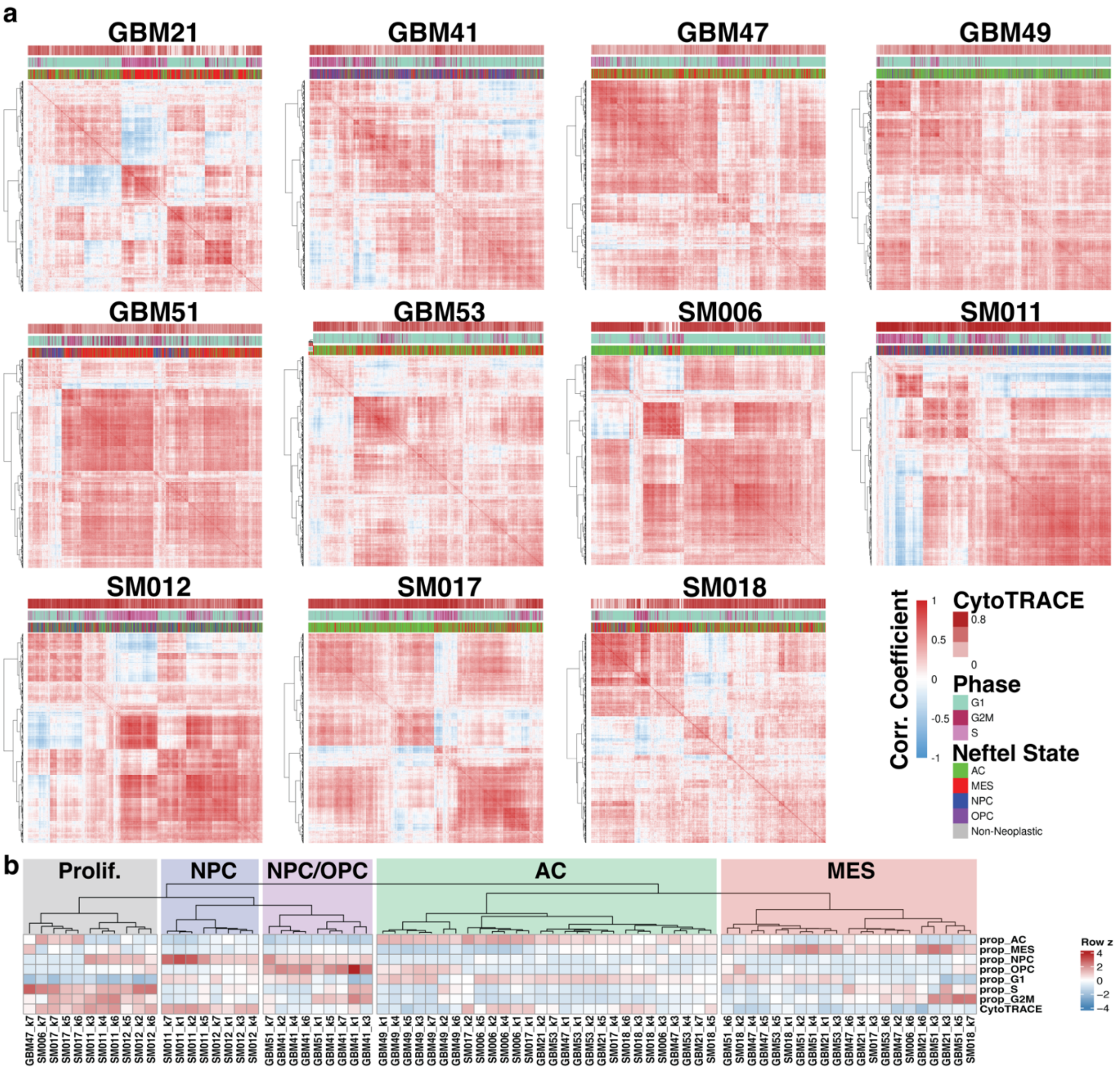

Supplementary Figure S3: Cell-drug connectivity using FDA-approved oncology drugs clusters patient GBM tumor cells reflecting patterns of GBM cell transcriptional state. a) Correlation matrices depicting pairwise Spearman correlations of single GBM tumor cells from each patient's tumor cells on drug connectivities with 63 FDA approved oncology drugs. Column annotations depict each cell's CytoTRACE score, cell cycle phase, and dominant Neftel et al transcriptional state. b) Heatmap depicting hierarchical clustering of all clusters k from all 11 patients on the relative proportions of cells within each GBM cell transcriptional state, the proportion of cells within each cell cycle phase, and mean CytoTRACE score. Source data provided within source data file.

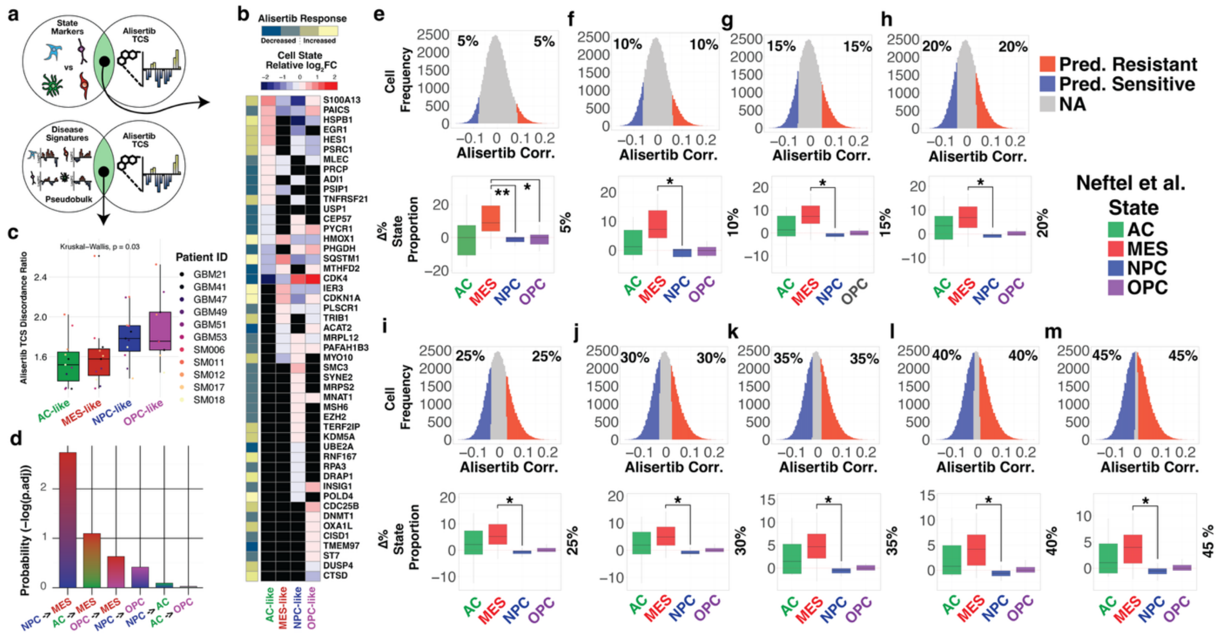

Supplementary Figure S4: Interrogation of an alisertib TCS using single-cell RNA sequencing indicates the predicted sensitivity of an NPC-like GBM cell state. a. Diagram describing interrogation of the L1000-derived alisertib TCS using GBM cell state markers as in (**b**) or by discordance ratio with GBM cell state disease signatures as in (**c**). **b.** Heatmap depicting differential expression of cell state markers that overlap with alisertib TCS genes. Cell color indicates the relative log_2FC of each gene, and black heatmap cells indicate a gene was not differentially expressed by a given cell state. Row annotation depicts magnitude and direction of gene expression changes of the alisertib TCS. **c.** Box plot of cell state disease signature discordance to the alisertib TCS by individual patient tumor. Cell state disease signatures were calculated using MAST to compare cells within each GBM cell state to non-neoplastic cell types within each individual. **d.** Bar plot depicting the pairwise post-hoc -log(p.adj) values of proportion change for each state combination as calculated from main Figure 3**c**. **e-m**. Histograms of patient GBM tumor cells colored by assigned alisertib TCS quantile-based sensitivity or resistance, paired with corresponding box plots of predicted proportion change for each Neftel et al GBM cell transcriptional state. For each quantile cut-off, the change in proportion was calculated by subtracting the proportion in the sensitive group from that of the resistant group. Post-hoc pairwise comparisons of relative state shifts were performed with the rstatix package using Wilcoxon rank-sum tests (two-sided), and the resulting p values were adjusted for multiple comparisons using the Holm method (*p.adj < 0.05, **p.adj <0.005).

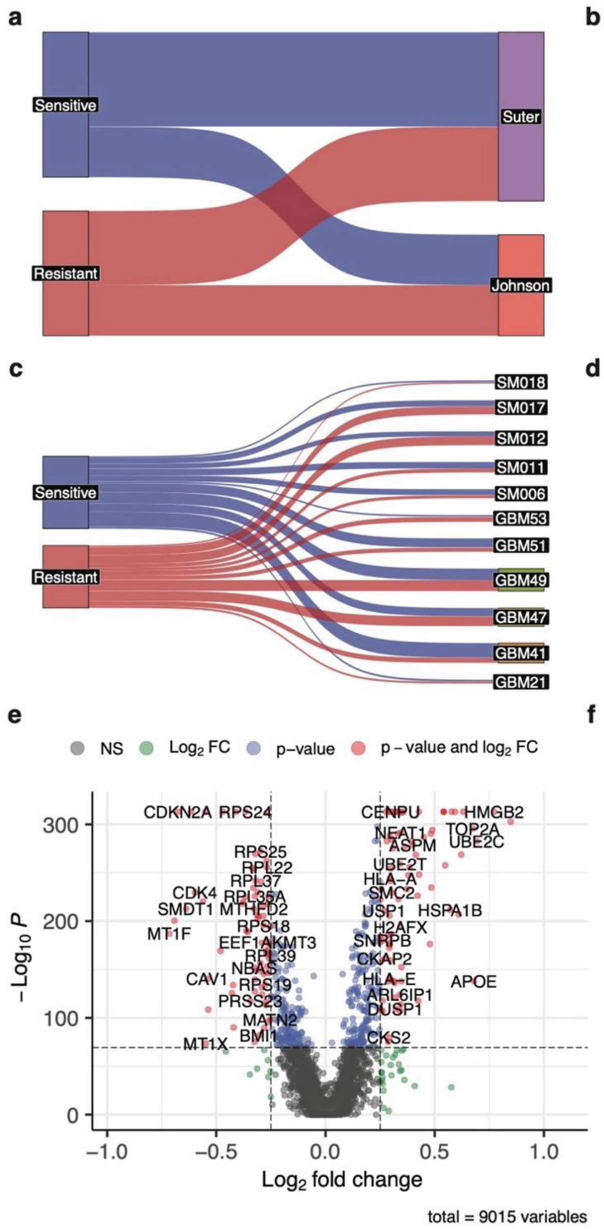

Supplementary Figure S5: scFOCAL predicts an alisertib-resistant cell population that spans the source dataset, initial occurrence or recurrence, individual patients, and cell cycle phase. a-d. Sankey diagrams depicting proportions of predicted sensitive and resistant cell populations across **(a)** source dataset, **(b)** initial occurrence or recurrence, **(c)** individual patients, and **(d)** cell cycle phase. **e.** Volcano plot of differentially expressed genes between scFOCAL-predicted resistant and sensitive cell populations. Genes with positive log_2FC represent those overexpressed in scFOCAL-predicted resistant tumor cells. **f.** Bar plot of mean module scores for scFOCAL-predicted resistant cell overexpressed genes as depicted in **e** of patient neoplastic cells grouped by predominant GBM cell transcriptional state. Error bars represent the 95% confidence interval. Data represented as an aggregate across 11 patient samples.

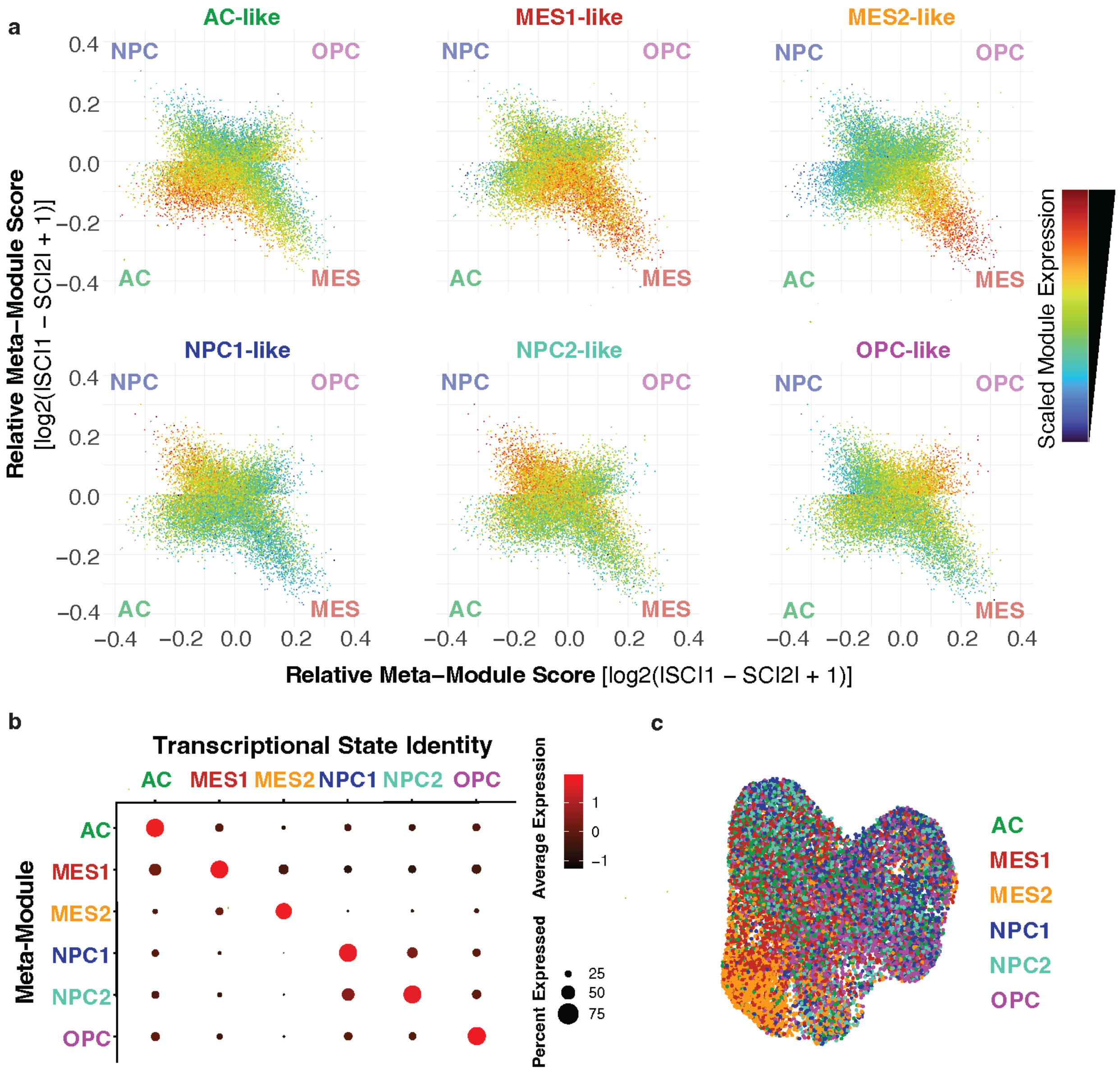

Supplementary Figure S6: GBM22 orthotopic xenografts are transcriptionally heterogeneous. a. Two-dimensional depiction of relative meta-module scores of GBM22 xenograft cells for the reported GBM cell transcriptional states defined by Neftel et al. (2019). Cells are colored by their enrichment for the six underlying GBM cell transcriptional state meta-modules. Each corner represents a transcriptional state meta-module. **b.** Dot plot of meta-module expression in GBM22 xenograft cells for GBM cell states grouped by assigned transcriptional state identity (6 states) based on predominantly enriched meta-module for each cell. The size of the dot encodes the percentage of cells within each transcriptional state with detected expression of the module, while the color depicts the mean expression level across all cells within each transcriptional state. **c.** UMAP of GBM22 orthotopic xenograft cells colored by assigned transcriptional state identity (6 states).

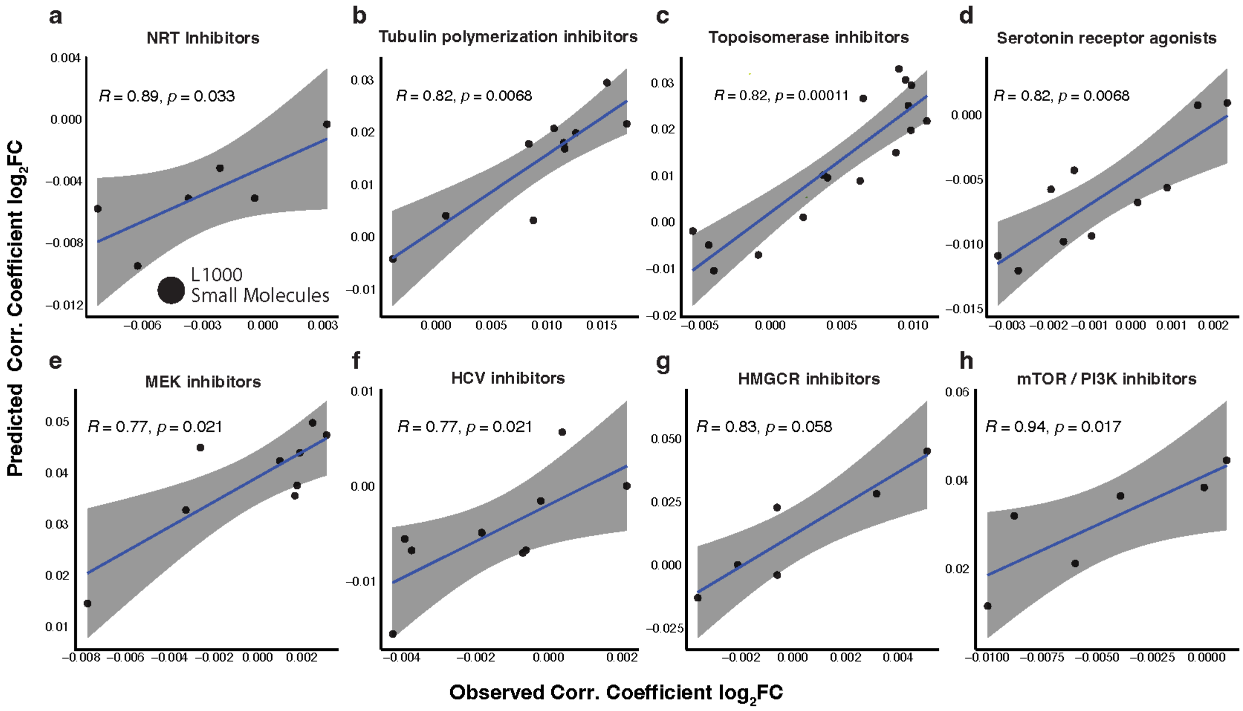

Supplementary Figure S7: In silico perturbation and predictive differential connectivity analysis models the drug-connectivity perturbation response to alisertib *in vivo*. a-h. Scatterplots of L1000 small molecule TCSs within individual compound classes depicting scFOCAL-predicted correlation shift (log_2FC) vs. observed correlation shift (log_2FC) in alisertib-treated xenografts. Differential small molecule correlations were calculated using *limma*. Resulting logFC values for each compound were compared across *in silico* and *in vivo* analyses using Spearman's ρ. Spearman correlation coefficient R (ρ) and p values were determined using a two-sided Spearman's correlation test (degrees of freedom=n-2). Shaded regions represent the 95% confidence interval of fitted regression lines. Source data are provided as a source data file.

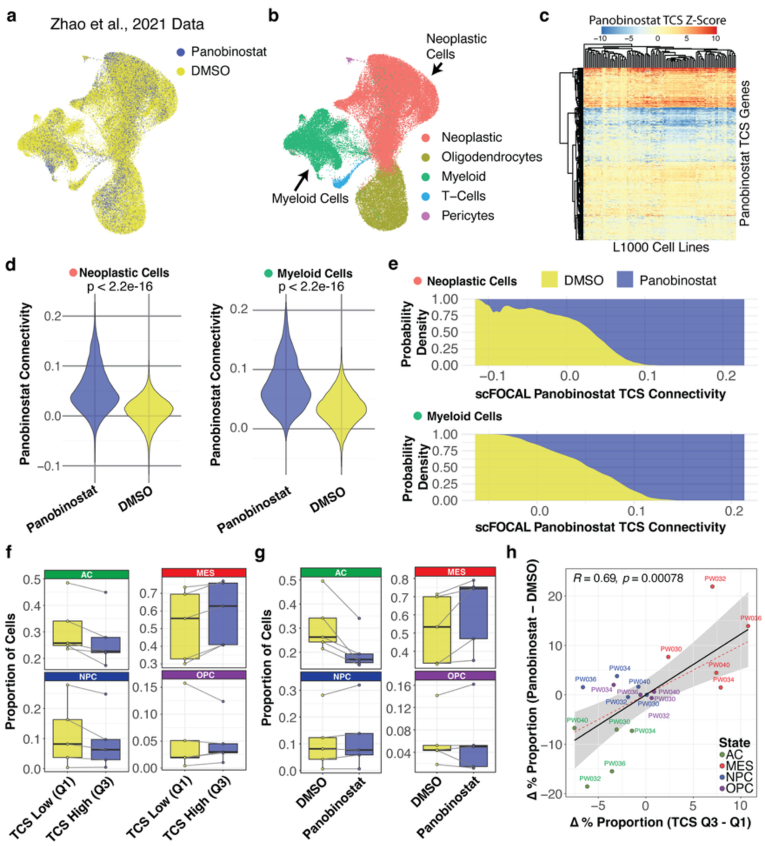

Supplementary Figure S8: scFOCAL connectivity analysis with an L1000-derived panobinostat TCS separates vehicle-treated from panobinostat-treated cells in both neoplastic and myeloid cell populations. a. UMAP of 62,250 single-cell transcriptomes from patient-derived acute slice culture samples obtained from Zhao et al., 2021, treated with DMSO or the HDAC inhibitor panobinostat (0.2 µM). **b.** UMAP of single-cell transcriptomes colored by discrete cell type. **c.** Heatmap showing z-scores of panobinostat-specific gene expression (vs. treatment naïve) for genes retained in the panobinostat TCS across cell lines tested in the L1000 dataset. **d.** Violin plots showing panobinostat TCS connectivity of panobinostat-treated vs. DMSO-treated cells in neoplastic and myeloid populations, respectively (Wilcoxon rank sum test with continuity correction, p-value < 2.2e-16). **e.** Area-normalized kernel-density estimate (KDE) plots of scFOCAL calculated panobinostat TCS connectivity colored by treatment with DMSO or panobinostat, across neoplastic and myeloid cell populations, respectively. **f.** Box plots of cell proportion for GBM cell transcriptional states, grouped on panobinostat TCS connectivity quartiles (high Q3 vs low Q1) (AC-like p = 0.062, MES-like p = 0.062, NPC-like p = 0.12, OPC-like p = 0.81, paired Wilcoxon). **g.** Box plots of cell proportion for each GBM cell transcriptional state, grouped by DMSO or panobinostat treatment (AC-like p = 0.062, MES-like p = 0.062, NPC-like p = 0.19, OPC-like p = 1, paired Wilcoxon) **h.** Scatterplot of proportion shifts (Q3 – Q1, Panobinostat – DMSO) predicted from Q1-Q3 comparisons, plotted against proportion shift between treatment groups. R (ρ) value determined using Spearman correlation.

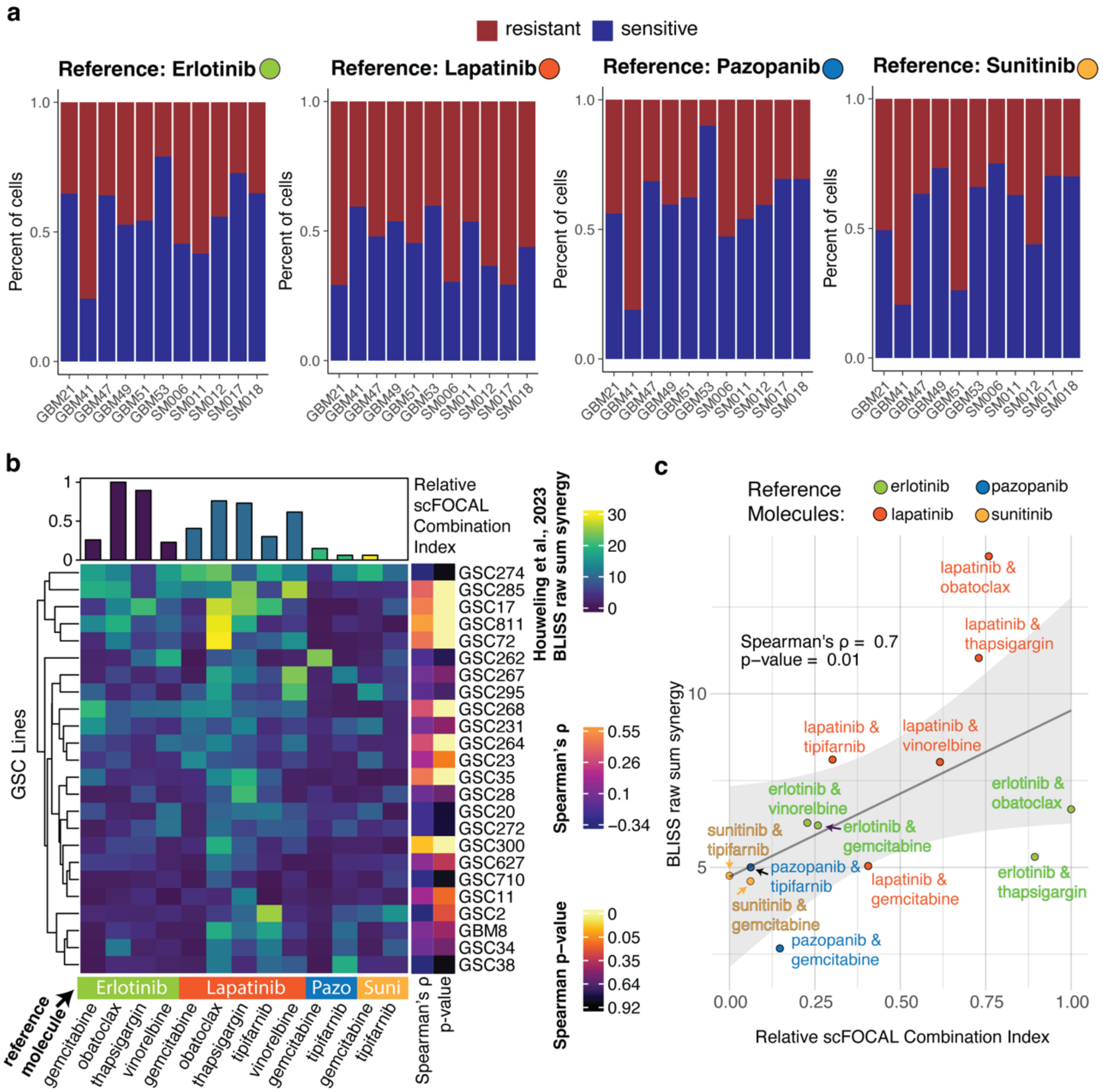

Supplementary Figure S9: scFOCAL combination scoring predicts relative synergy of small molecule combinations *in vitro*. a. Stacked bar plots depicting proportion of cells predicted to be sensitive or resistant across individual patient GBM tumors to reference small molecules erlotinib, lapatinib, pazopanib, and sunitinib respectively. **b.** Heatmap of BLISS raw sum synergy of small molecule combinations obtained from Houweling et al., 2023. Top bar plot annotation depicts scFOCAL calculated combination score using labeled reference molecules depicted on the bottom annotation bar. Sidebar annotations depict Spearman's ρ and p-value between scFOCAL combination score and observed BLISS raw sum synergy for each individual cell line. **c.** Scatterplot of scFOCAL calculated combination score and mean BLISS raw sum synergy across all cell lines tested for small molecule combinations screened in Houweling et al., 2023 (Spearman's ρ = 0.7, p-value = 0.01). Points are colored by the reference molecule used for combination scoring. Shaded gray area around the line of best fit depicts standard error.

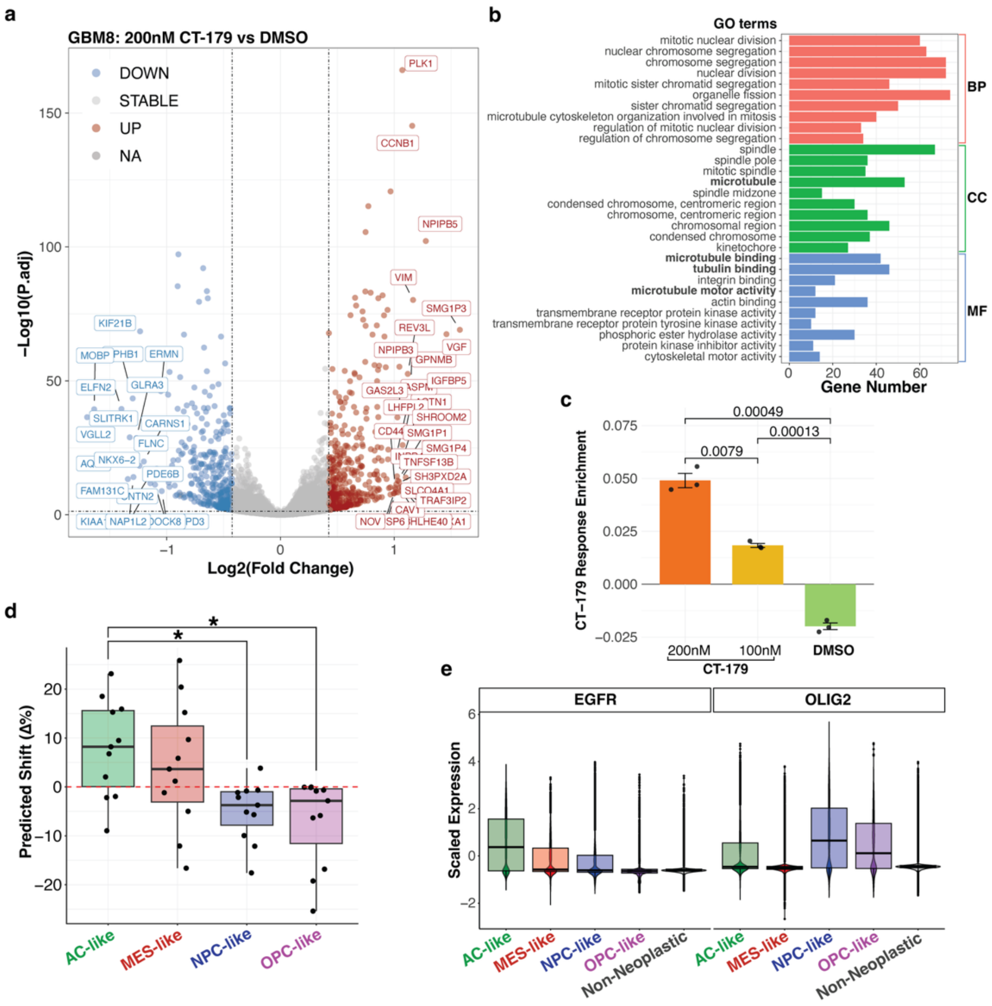

Supplementary Figure S10: Generation of a GBM CT-179 transcriptional response signature. **a.** Volcano plot of differential expression between 200nM CT-179 and vehicle treated GBM8 cells calculated via DESeq2 (p.adj < 0.05, |LFC| > 2SD+mean(LFC)). **b.** Barplot depicting gene ontology analysis of the filtered CT-179 transcriptional response signature. Bars are colored by the respective ontology used (BP = Biological Process; CC = Cellular Component; MF = Molecular Function). Selected GO terms highlighted include 'microtubule', 'microtubule binding', 'tubulin binding', and 'microtubule motor activity'. **c.** Barplot of singscore-calculated enrichment for the directional CT-179 transcriptional response signature in 200nM CT-179 treated, 100nM CT-179 treated, and vehicle-treated GBM8 cells after 24 hours. Significance bars depict p-value following pairwise t-tests (n = 3 per treatment group). **d.** Box plot depicting per-patient difference in proportions of cells in each GBM cell transcriptional state in CT-179 resistant vs sensitive populations. Each dot represents the difference for an individual patient (n=11). Boxplot elements represent the median (center-line), quartiles (box limits), and 1.5x interquartile range (whiskers). Adjusted p-value from a two-sided Wilcoxon signed-rank test with Bejamini Hochberg correction (AC vs NPC p.adj=0.012; AC vs OPC p.adj=0.012) **e.** Box and violin plots depicting the expression of EGFR and OLIG2 in patient GBM tumor cells grouped by assigned Neftel et al. GBM cell transcriptional state.
