# Supplementary material for: Drug and single-cell gene expression integration identifies sensitive and resistant glioblastoma cell populations
Source: Nat Commun. 2026 Jan 7;17:99. doi: 10.1038/s41467-025-67783-5 (PMC12780015; doi:10.1038/s41467-025-67783-5)
Supplement: Supplementary file 2 — Description of Additional Supplementary Files [file 41467_2025_67783_MOESM2_ESM.pdf]

Title: Supplementary Data 1

Description: Comma-separated table of MAST differential expression testing results between discrete cell types identified in patient GBM scRNAseq data.

Title: Supplementary Data 2

Description: Comma-separated table of MAST differential expression testing results between GBM tumor cells within each Neftel et al. transcriptional state and non-tumor cell types captured from the tumor microenvironment.

Title: Supplementary Data 3

Description: Comma-separated table of MAST differential expression testing results between GBM tumor cells within each Neftel et al. transcriptional state, within each individual patient tumor, respectively, compared to all non-tumor cell types captured from the tumor microenvironment.

Title: Supplementary Data 4

Description: Comma-separated table of calculated reversal scores for each compound in the L1000 dataset against each of the aggregate (across all patients) disease signatures calculated for each Neftel et al. GBM cell transcriptional state.

Title: Supplementary Data 5

Description: Excel .xlsx file of results of differential drug connectivity between alisertib-resistant and sensitive cell populations determined in silico (Tab 1: patient_DrugDiscordancelimma_result) and in vivo (Tab 2: pdx_DrugDiscordancelimma_result).

Title: Supplementary Data 6

Description: Excel .xlsx file of results of predicted CT-179 sensitive and resistant cell differential drug connectivity analysis, mean CT-179 resistant cell connectivity values, and derived scFOCAL combination index scoring
